# Endovascular repair of a bacillus Calmette-Guérin mycotic aortic aneurysm

**DOI:** 10.1016/j.jvscit.2022.08.018

**Published:** 2022-08-31

**Authors:** Nader Tehrani, Sara Will, Pegge Halandras

**Affiliations:** Division of Vascular Surgery, Edward Hines Jr Veterans Affairs Hospital, Hines, IL

**Keywords:** BCG, EVAR, Mycotic aneurysm

## Abstract

Bacillus Calmette-Guérin (BCG) is an attenuated form of *Mycobacterium bovis* used for intravesical treatment of bladder carcinoma. Aortic infection has been rare. In the present report, we have described the case of a patient with an infrarenal mycotic aortic pseudoaneurysm and para-aortic abscess after intravesical bacillus Calmette-Guérin and cystectomy. Sampling of the abscess demonstrated acid-fast bacilli. Given the hostile anatomy of the abdomen, he was offered endovascular aortic repair. A thoracic endograft was used to cover the lesion. The patient was discharged on postoperative day 2 without incident. He was seen at 1 and 6 months with resolution of his pseudoaneurysm found on the imaging studies. The technique shows promise for stabilizing such lesions with close surveillance.

Bacillus Calmette-Guérin (BCG) is a live attenuated strain of *Mycobacterium bovis* widely used for the treatment of bladder carcinoma. Several randomized controlled trials have demonstrated a reduced incidence of recurrence of high-risk superficial transitional cell carcinoma with BCG therapy.^1^^,^^2^ Intravesical treatment has been considered relatively safe, with the most common risks being fever, pelvic discomfort, and hematuria.^3^ Although infections due to BCG dissemination have been rare, cases have been reported of granulomatous hepatitis, pneumonitis, and prostatitis occurring in <1% of treated patients.^3^ Regarding vascular infections, <30 cases of BCG-associated aortitis and resulting mycotic aneurysms have been reported.^3^, ^4^

The standard treatment of mycotic aortic aneurysms involves open repair of the aorta with either in situ or extra-anatomic revascularization, debridement of the retroperitoneum, and a prolonged antibiotic regimen. Perioperative mortality has been reported to approach 20% for open surgery in this complex patient population.^5^, ^6^, ^7^ Endovascular repair has been described for prohibitive surgical candidates and unstable patients, with some early success; however, the risks of relapse and persistent infection remain.^8^

We have reported the case of a mycotic infrarenal aortic aneurysm in a 73-year-old man who had received intravesical BCG therapy for bladder cancer. Given the low virulence of *M. bovis*, the presence of a complex urologic surgical history, and the patient’s overall frailty, an endovascular approach was chosen for treatment. The patient provided written informed consent for the report of his case details and imaging studies.

## Case report

**Fig 1:**
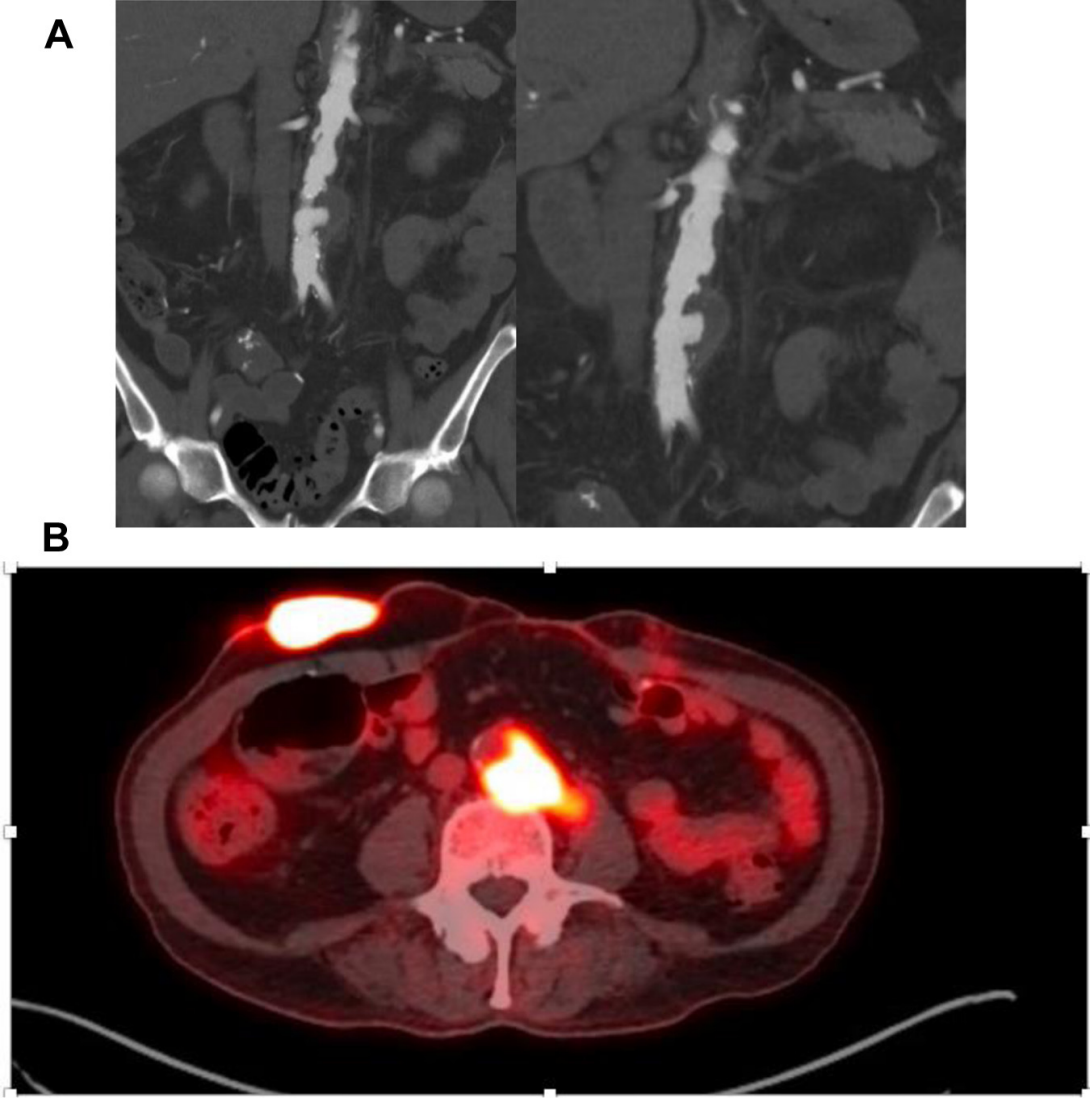
**A,** Infrarenal aortic saccular aneurysm. **B,** The lesion and associated iliopsoas abscess demonstrated positron emission tomography avidity.

The patient was seen by the vascular service after inpatient admission for abdominal pain and leukocytosis. His history was notable for bladder carcinoma that had been treated with two cycles of intravesical BCG. Follow-up cystoscopy with biopsy demonstrated persistent in situ disease with no locoregional or metastatic spread. He had ultimately undergone cystoprostatectomy with creation of an ileal conduit. His postoperative course had been complicated by a bowel injury requiring reexploration, cecectomy, and a diverting ileostomy with eventual reversal. Several months after his abdominal operations, he had presented to the Veterans Affairs hospital with abdominal pain, low-grade fevers, and leukocytosis. Computed tomography (CT) demonstrated an infrarenal aortic pseudoaneurysm with a para-aortic and iliopsoas abscess (Fig 1). A fine needle aspirate and sputum cultures were positive for acid-fast bacilli, raising concerns for disseminated BCG infection. Intravenous isoniazid, rifampin, and ethambutol were initiated. The standard fourth antibiotic for tuberculosis, pyrazinamide, was held owing to natural resistance in *M. bovis.* The urology, infectious disease, and interventional radiology services were all consulted for discussion of the definitive treatment. Given his hostile abdominal anatomy and an ∼50-lb weight loss, an open approach was deemed prohibitive. A retroperitoneal approach was discussed with the urology service, which stated that disseminated BCG infection will result in extensive inflammatory changes and scarring to the retroperitoneum. In addition, the transposed ureter to the ileal conduit would have a significant risk of injury owing to the abnormal course, resulting in a permanent nephrostomy tube for the patient. The focal nature of the lesion and distance from the renal arteries to the iliac bifurcation provided an opportunity for a single tubular endograft piece. Thus, we decided to implant a thoracic endograft to prevent future degeneration and rupture. In addition, a Dacron endograft was chosen because it could be soaked in rifampin, which has ideal activity against mycobacterial species.

## Treatment

**Fig 2:**
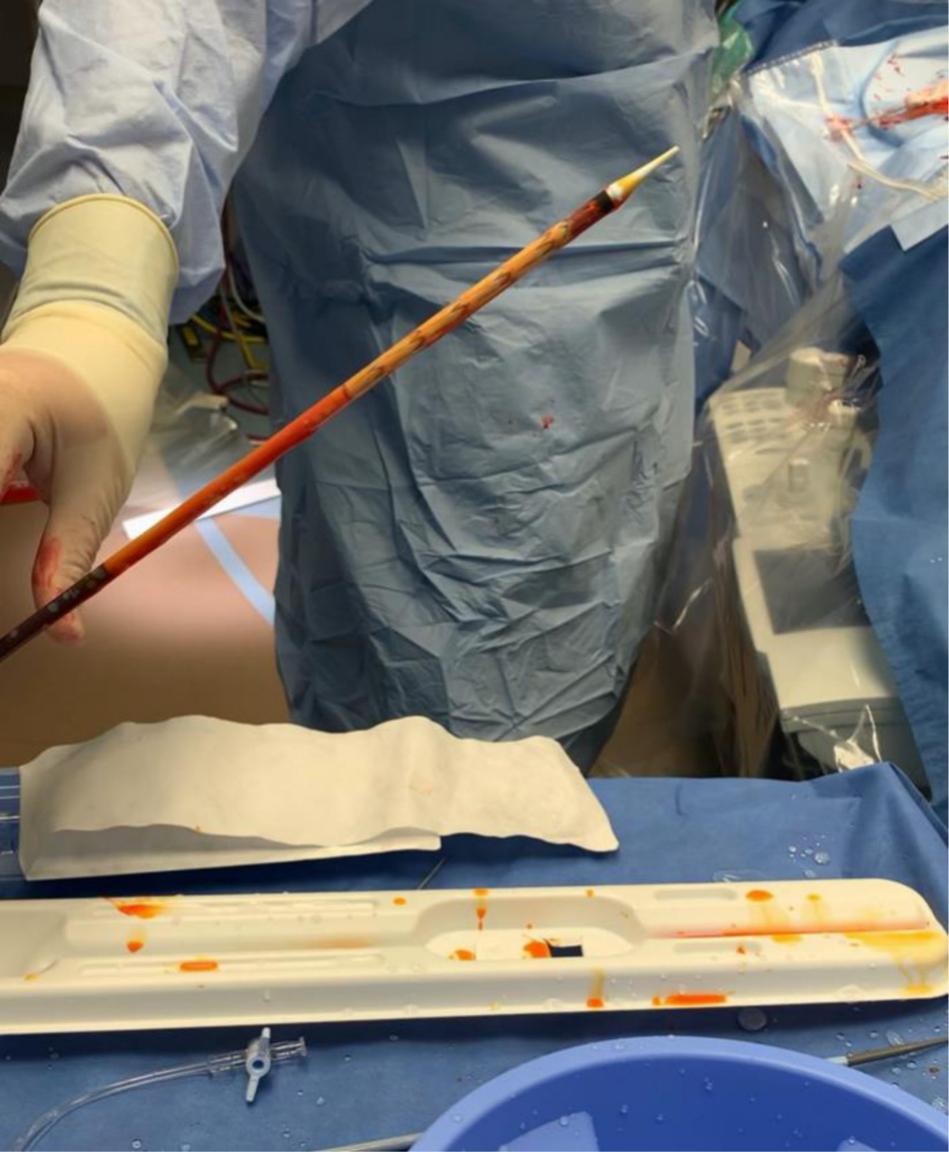
The Dacron-based thoracic endograft was flushed with rifampin solution through the device port until staining of the fabric was visible.

**Fig 3:**
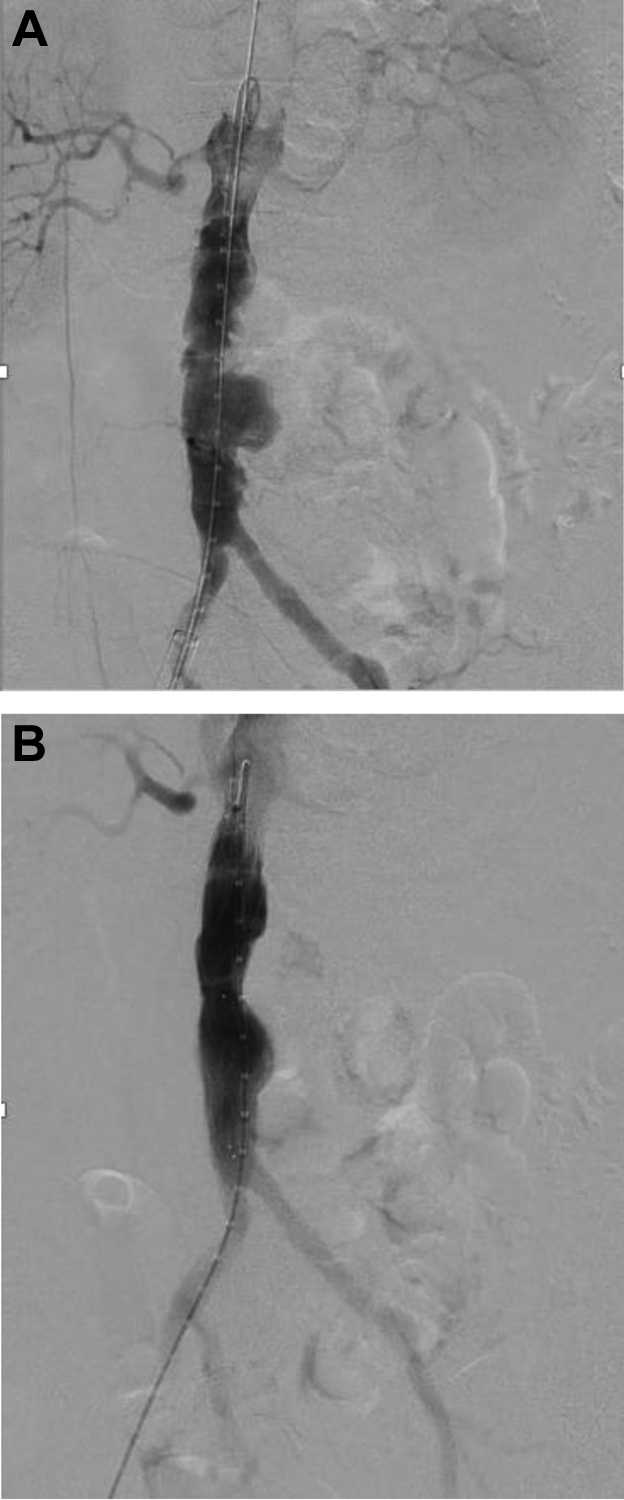
**A,** Preintervention aortography. **B,** Successful deployment of thoracic endograft with at least one stent graft overlap distally.

Endovascular repair of the mycotic pseudoaneurysm was performed with the patient under general anesthesia. The procedure was performed through a single access site in the right common femoral artery. Access was achieved percutaneously with the deployment of two preclosure sutures. An 18F DrySeal sheath (W.L. Gore & Associates, Flagstaff, AZ) was advanced into the artery over a stiff wire. A pigtail catheter was passed over a guidewire into the aorta and used for intraoperative imaging. A 22 × 94-mm Navion endograft (Medtronic, Dublin, Ireland) was chosen for the repair before company recall of the device. A solution of 150 mL of saline mixed with 600 mg of rifampin was used to flush the Dacron-based endograft. Multiple flushes were performed until the entirety of the endograft fabric appeared visibly stained (Fig 2). Aortography revealed the lesion clearly and confirmed the length from the renal arteries to the aortic bifurcation. The endograft was deployed across the lesion, with care taken to achieve adequate overlap distally without encroaching on the iliac orifices. Gentle postdilation was performed with a molding balloon. Completion angiography revealed excellent coverage of the lesion (Fig 3). The sheath was removed, and the preclosure sutures were used to achieve hemostasis without incident. The patient did well postoperatively. A retroperitoneal drain was placed by interventional radiology to drain the associated abscess. The patient was discharged home on the second postoperative day. The drain was removed after a CT scan at 3 weeks postoperatively had demonstrated resolution of the abscess.

**Fig 4:**
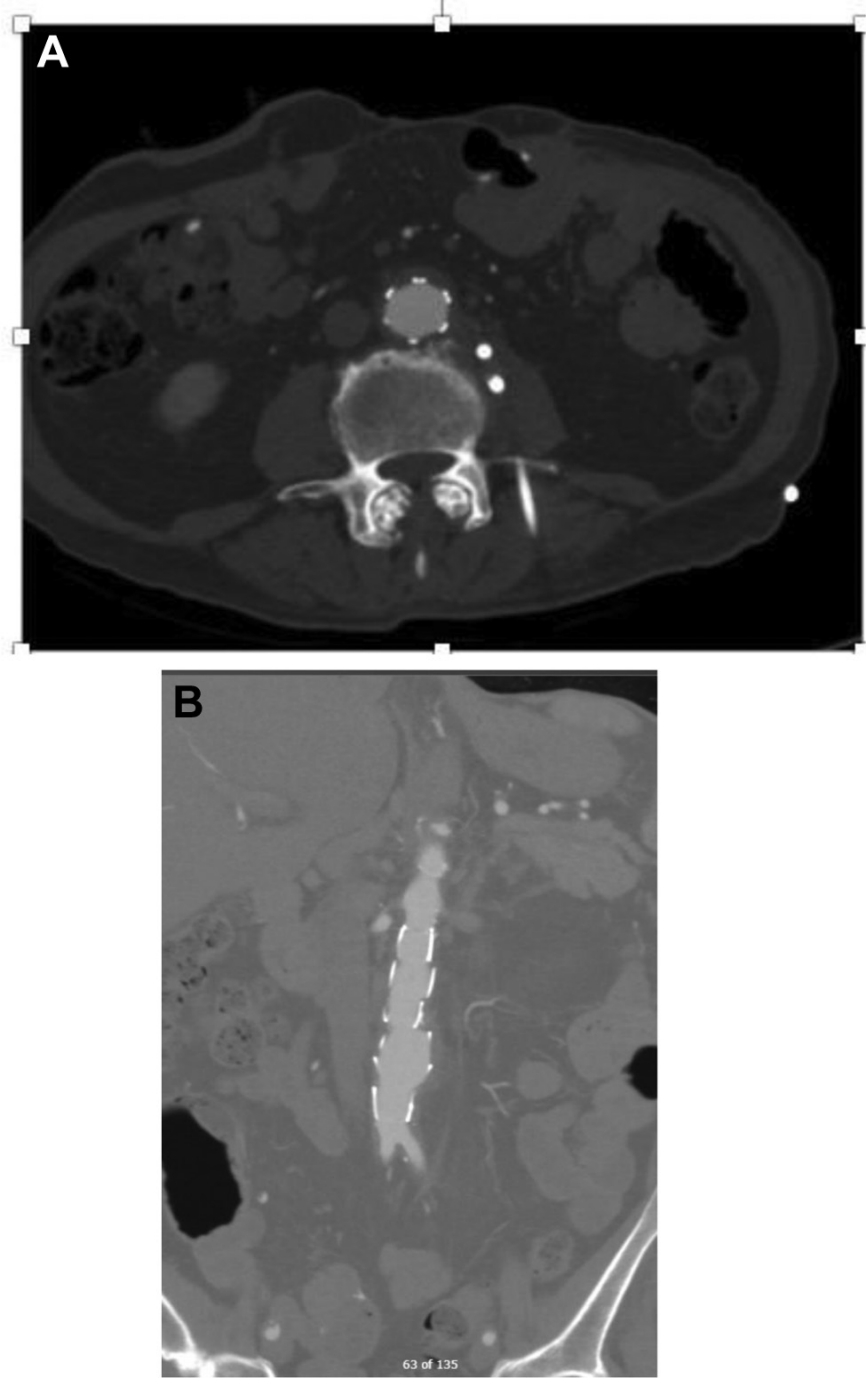
**A,** Postoperative computed tomography (CT) scan with good apposition of the endograft and associated interventional radiology–placed retroperitoneal drain. **B,** Follow-up imaging at 6 months with no radiographic evidence of recurrence.

The patient was seen by infectious disease at 3 months with no signs of disseminated infection present. He continued receiving isoniazid and rifampin for chronic suppression with the plan for the use of an indefinite antibiotic regimen. A CT scan at 6 months demonstrated thrombosis of the aneurysm sac with no recurrent retroperitoneal abscess (Fig 4). The patient was clinically doing well, gaining weight, and had had negative sputum cultures, indicating resolution of the disseminated infection.

## Discussion

Our review of the literature revealed <30 reported cases of mycotic aneurysms secondary to BCG-related *M. bovis*.^9^, ^10^, ^11^ Similar to prior reports, our patient had presented with a protracted infectious course consisting of months of fevers, weight loss, and abdominal pain. Other complications that have been described include aneurysm rupture and aortoenteric fistula.^9^ A delay in diagnosis is unsurprising given that BCG-related adverse effects have been cited at <5%, with only a 0.4% rate of life-threatening sepsis.^3^ Studies of the repair of both aortic and peripheral aneurysms have mostly described open in situ replacement or bypass. Given the low virulence of BCG infection, in situ replacement with a prosthesis has been described for most cases.^9^ Only three endovascular cases for exclusion of mycotic aortic aneurysms were identified in our review, with one patient dying of postoperative myocardial infarction and another lost to follow-up at 8 months after treatment.^12^^,^^13^ The third report had documented successful exclusion of the abdominal aneurysm with an endograft. However, the patient had experienced a recurrence with a fistulizing iliopsoas abscess to the aneurysm sac at 30 months, requiring graft excision and extra-anatomic bypass.^14^ These reports had not described antibiotic impregnation of the endografts. In our patient, the associated para-aortic abscess was well controlled with image-guided drainage.

Understandably, a standard surveillance algorithm has not been established for such a rare occurrence. The reported studies for in situ repair have recommended annual CT scans and have even suggested biannual imaging early on for surveillance of recurrence.^9^ For our patient, surveillance CT was planned for every 6 months for the first 2 years. Similarly, no definitive criteria have been established for the duration of an antituberculosis antibiotic regimen, especially for patients treated without open resection and debridement.

## Conclusions

Mycotic aortic aneurysm is a rare complication of urothelial BCG treatment. The few available studies have mostly described open repair with in situ prosthetic revascularization. Despite the acceptable results, an open approach could be prohibitive for many of these patients who have undergone complex urologic resections and reconstructive procedures. In our report, we have described endovascular repair with image-guided drainage and a long-term antibiotic regimen as an alternative treatment modality with early term infection resolution and suitable exclusion of the lesion in a high-risk patient.
